# Real-world clinical effectiveness and safety of vedolizumab and anti-tumor necrosis factor alpha treatment in ulcerative colitis and Crohn’s disease patients: a German retrospective chart review

**DOI:** 10.1186/s12876-020-01332-w

**Published:** 2020-07-08

**Authors:** Ulf Helwig, Michael Mross, Stefan Schubert, Heinz Hartmann, Alina Brandes, Dara Stein, Christian Kempf, Jana Knop, Sarah Campbell-Hill, Robert Ehehalt

**Affiliations:** 1 Gastroenterology Private Practice, Neue Donnerschweer Str. 30, 26123 Oldenburg, Germany; 2 University of Kiel, Kiel, Germany; 3 Gastroenterology Private Practice, Berlin, Germany; 4 Gastroenterology Private Practice, Herne, Germany; 5 Takeda Pharma Vertrieb GmbH & Co. KG, Berlin, Germany; 6 Evidera, London, UK; 7 Takeda Pharmaceuticals International AG, Zurich, Switzerland; 8 Takeda International – UK Branch, London, UK; 9 Gastroenterology Outpatient Clinic, Heidelberg, Germany

**Keywords:** Inflammatory bowel disease, Crohn’s disease, Ulcerative colitis, Outcomes research, Vedolizumab, Real-world evidence

## Abstract

**Background:**

Real-world comparisons of biologic treatment outcomes for ulcerative colitis (UC) or Crohn’s disease (CD) patients are limited. We sought to evaluate the real-world effectiveness of vedolizumab (VDZ) and anti-tumor necrosis factor alpha (anti-TNFα) in UC and CD patients in Germany.

**Methods:**

A retrospective chart review (15 sites) investigated UC and CD patients who were biologic-treatment naïve (biologic-naïve) or had received no more than one prior anti-TNFα before initiating treatment with VDZ or anti-TNFα between 15 July 2014 and 20 October 2015. Kaplan-Meier analyses assessed time to first chart-documented clinical remission (CR) and symptom resolution (UC: rectal bleeding [RB], stool frequency [SF]; CD: abdominal pain [AP], liquid stools [LS]) and outcome duration.

**Results:**

A total of 133 UC (76 VDZ; 57 anti-TNFα) and 174 CD (69 VDZ; 105 anti-TNFα) patients were included. By Week 26, estimated cumulative rates of patients achieving CR or symptom resolution with VDZ vs anti-TNFα treatment were for UC: CR, 53.7% vs 31.7%; RB, 66.8% vs 55.8%; and SF, 59.8% vs 50.7%, respectively; and for CD: CR, 14.4% vs 32.8%; AP, 62.5% vs 56.0%; and LS, 29.9% vs 50.3%, respectively. Outcomes were sustained similarly between treatments, except RB (VDZ vs anti-TNFα: median 38.1 vs 15.1 weeks, *P* = 0.03). Treatment-related adverse events occurred in 5.3% vs 7.0% (UC) and 8.7% vs 19.0% (CD) of VDZ vs anti-TNFα patients, respectively.

**Conclusions:**

Although there were differences in CR, symptom resolution, and safety profiles, real-world data support both VDZ and anti-TNFα as effective treatment options in UC and CD.

## Background

Ulcerative colitis (UC) and Crohn’s disease (CD) both originate from dysregulation of the immune system and are the most common types of inflammatory bowel disease (IBD), with prevalence increasing in Germany and worldwide [1, 2]. Recent estimates of prevalence in Germany were 412 (95% confidence interval [CI], 389–436) cases of UC and 322 (95% CI, 302–346) cases of CD per 100,000 persons [2]. Both UC and CD are chronic diseases that often require life-long treatment and frequent hospitalization, resulting in reduced patient quality of life and substantial healthcare resource utilization [3, 4].

Patients with moderately or severely active IBD who have had an inadequate disease response, lost response, or were intolerant to a conventional treatment such as a corticosteroid, aminosalicylate, and/or immunomodulatory drug may be treated with a biologic agent, such as a tumor necrosis factor alpha (anti-TNFα) antagonist (adalimumab, infliximab, or golimumab [approved in Europe for UC only]), ustekinumab, vedolizumab, or with the oral small molecule janus kinase (JAK) inhibitor tofacitinib (approved for UC only). Meta-analyses have shown that in patients with IBD, anti-TNFα therapy in comparison with placebo results in higher likelihood of induction of remission and response as well as maintenance of remission and response [5, 6]. However, up to 30% of patients do not respond to anti-TNFα treatment (primary non-response) and 23 to 46% of patients lose response over time (secondary loss of response) [7]. High treatment failure rates indicate a need for other first- and second-line biologic treatment options to improve the management and outcomes of patients with UC or CD [8–12].

Vedolizumab, a gut-selective α_4_β_7_ integrin antagonist, was approved by the European Medicines Agency (EMA) in May 2014 for the treatment of moderately to severely active UC and CD. Vedolizumab blocks the binding of integrin α_4_β_7_ on lymphocytes to mucosal vascular addressin cell adhesion molecule 1 (MAdCAM-1) on gut endothelial cells, resulting in reduced lymphocyte trafficking into gut tissue. In the phase 3, randomized, double-blind, placebo-controlled GEMINI trials, vedolizumab effectively improved clinical remission (CR) and increased symptom resolution in patients with active UC (GEMINI 1) and CD (GEMINI 2 and 3) [13–15]. Subgroup analyses of the GEMINI trials showed more pronounced treatment effects in anti-TNFα–naïve patients than in patients previously treated with anti-TNFα therapy [13, 14]. Real-world evidence also supports the greater effectiveness of vedolizumab in anti-TNFα–naïve patients [16–19] and can be utilized to compare the effectiveness and safety of vedolizumab and anti-TNFα and help inform clinical treatment choices. This is particularly important for key patient sub-groups, such as those naïve or refractory to biologic treatments, for whom gastroenterologists must select the most appropriate treatment to achieve optimal outcomes.

This study aimed to assess the real-world effectiveness and safety of vedolizumab and anti-TNFα in patients with UC or CD treated at multiple centers in Germany, shortly after vedolizumab became available. Patients were either biologic treatment naïve or had received no more than one prior anti-TNFα therapy.

## Methods

### Study design

A retrospective, multicenter medical chart review study was conducted between June 2016 and January 2017. The study evaluated patients with IBD initiated with vedolizumab or an anti-TNFα between 15 July 2014 and 20 October 2015 at 15 sites in Germany. The 4 university and 11 private practice study sites were geographically dispersed and of varied sizes. The study was approved by the local ethics committee at each participating site (Additional file 1). All patients alive at the time of chart abstraction (99% of patients) signed an informed consent form prior to participation in this study.

Adult patients (≥18 years of age) with UC or CD who were biologic-naïve or who had received no more than one prior anti-TNFα and initiated “index treatment” with either vedolizumab or an anti-TNFα (infliximab-originator, infliximab-biosimilar, adalimumab, or golimumab [UC only]) between 15 July 2014 and 20 October 2015 were eligible. Patients were excluded if their index treatment was administered as part of a clinical trial, if they had received more than one anti-TNFα treatment before they initiated index treatment, or if they had received prior treatment with biologic agents for conditions other than IBD.

The post-index follow-up period was defined as the time period between index treatment initiation and the earliest of date of chart abstraction initiation, date of death, or date of last contact with the site.

### Study data

Data on patient demographics, clinical history, and treatment history were collected prior to index treatment in the time period beginning on the date of diagnosis of UC or CD and ending 1 day before the date of index vedolizumab or anti-TNFα treatment initiation during the eligibility period. Data collected included age, sex, disease duration, comorbidities, and prior medical and surgical treatment. Data were also collected on disease location, disease activity, and concomitant non-biologic therapies at index treatment initiation. Due to variability in the timing of real-world clinical appointments and the completeness of records, a “window” for the evaluation of baseline disease activity was applied, using the chart-recorded patient assessment closest to Day 0 (index treatment initiation) from Day − 182 to Day 0. Following index treatment, data were collected on treatment patterns, treatment effectiveness, adverse events (AEs), and mortality. Patients were followed-up from index treatment initiation to the first of index treatment discontinuation, death, loss to follow-up, or chart abstraction.

Study outcomes (events) of interest included the incidence of clinical remission and, measured separately, symptom remission within 6 months (26 weeks) from index treatment initiation. These outcomes were selected after considering the Selecting Therapeutic Targets in Inflammatory Bowel Disease (STRIDE) guidelines, which recommend that clinical/patient-reported outcome remission should be the treatment target in both UC and CD [20]. Although STRIDE recommends that these outcomes should be evaluated in combination with endoscopic remission, the limited availability of endoscopic data precluded evaluation of a composite endpoint in the current study.

In patients with UC, several events of interest were analyzed: CR and resolution of rectal bleeding (RB) and stool frequency (SF) symptoms. Clinical remission was defined as a total Mayo score ≤ 2 with no individual subscore > 1 or a partial Mayo score ≤ 2 with no individual sub-score > 1, RB resolution was defined as a Mayo RB sub-score of 0, and SF resolution was defined as a Mayo SF sub-score of 0 or 1. In patients with CD, several events of interest were analyzed: CR and resolution of abdominal pain (AP) and liquid stool (LS) symptoms. Clinical remission was defined as a Harvey-Bradshaw Index (HBI) score < 5, AP resolution was defined as an AP score of 0 (no pain) or 1 (mild pain), and LS resolution was defined as ≤1.5 liquid or very soft stools per day.

### Statistical analysis

Analyses were conducted on the full effectiveness analysis set, defined as patients with chart-recorded assessments both at baseline (between Day − 182 and Day 0) and after their index event (initiation of index treatment [vedolizumab or anti-TNFα] during the study eligibility period). Results were stratified by indication (UC or CD) and index treatment (vedolizumab or anti-TNFα), and sub-analyses were further stratified by prior biologic treatment history (biologic-naïve vs one prior anti-TNFα).

Descriptive statistics (mean, median, range, standard deviation [SD], and 95% CI) were computed for continuous variables. Categorical variables were described by frequency and percentages. Partially missing dates of UC or CD diagnosis and AEs were imputed; a missing month was imputed as 01 [January] and a missing day was imputed as 1 [first day of month]). All AEs were included in the analyses, even if the imputed date was before the index date. No other imputation of missing data was performed. The number of patients with missing records for any given variable is reported. Percentages were calculated using the total number of patients with available data as the denominator.

To assess clinical effectiveness, time-to-event was analyzed and the cumulative rate of patients experiencing the respective events by Week 26 was estimated using a nonparametric, stratified Kaplan-Meier (KM) approach to account for variability in patient follow-up and timing of outcome events. Patients in CR or with symptom resolution at baseline (based on most recent chart-documented assessment before index event from Day − 182 to Day 0) and who were not switched to index therapy due to “lack of/incomplete UC or CD response to prior therapy” were left censored at Day 0. Patients who discontinued their index treatment (for any reason) before the occurrence of the event of interest were censored at the date of index treatment discontinuation. Patients who did not present the event of interest during the post-index follow-up period and who did not discontinue the index treatment were censored at the date of last available information.

The duration of each outcome was also derived. Patients who did not present the event of interest (clinical or symptom remission) between Day 0 and Day 182 were left censored. For patients who presented with the event of interest, the time from the first chart record of the outcome having been achieved to the first chart record of the outcome no longer being achieved (defined as the inverse of the definition of each outcome) or index treatment discontinuation due to lack of effectiveness was assessed. Patients who did not meet the inverse definition of each outcome and who did not discontinue the index treatment were censored at the date of last available information.

For each outcome analysis, the number of patients at risk at index treatment initiation was dependent on the number of patients that were left censored. Consequently, since the number of patient “responders” at baseline varied between different assessed outcomes, the number of patients at risk at Day 0 was also variable. Kaplan-Meier survival curves by strata were compared using the log-rank test.

The number and percentage of patients with an AE were reported and, to account for variability in patient follow-up, AEs were also reported as incidence per 100 patient-years of exposure (number of patients experiencing an AE of interest divided by total time in years patients were at risk, multiplied by 100). Time at risk was defined as the duration between the date of index treatment initiation to the date of the first event of interest, 18 weeks post-treatment discontinuation (5 half-lives of vedolizumab) [21], or date of last observation (whichever occurred first). If the imputed AE date was prior to the index date, the time at risk was defined as 0. All analyses were conducted using SAS version 9.4 software (SAS Institute, Cary, NC, USA).

## Results

### Patient baseline clinical and disease characteristics

#### UC patients

**Fig. 1:**
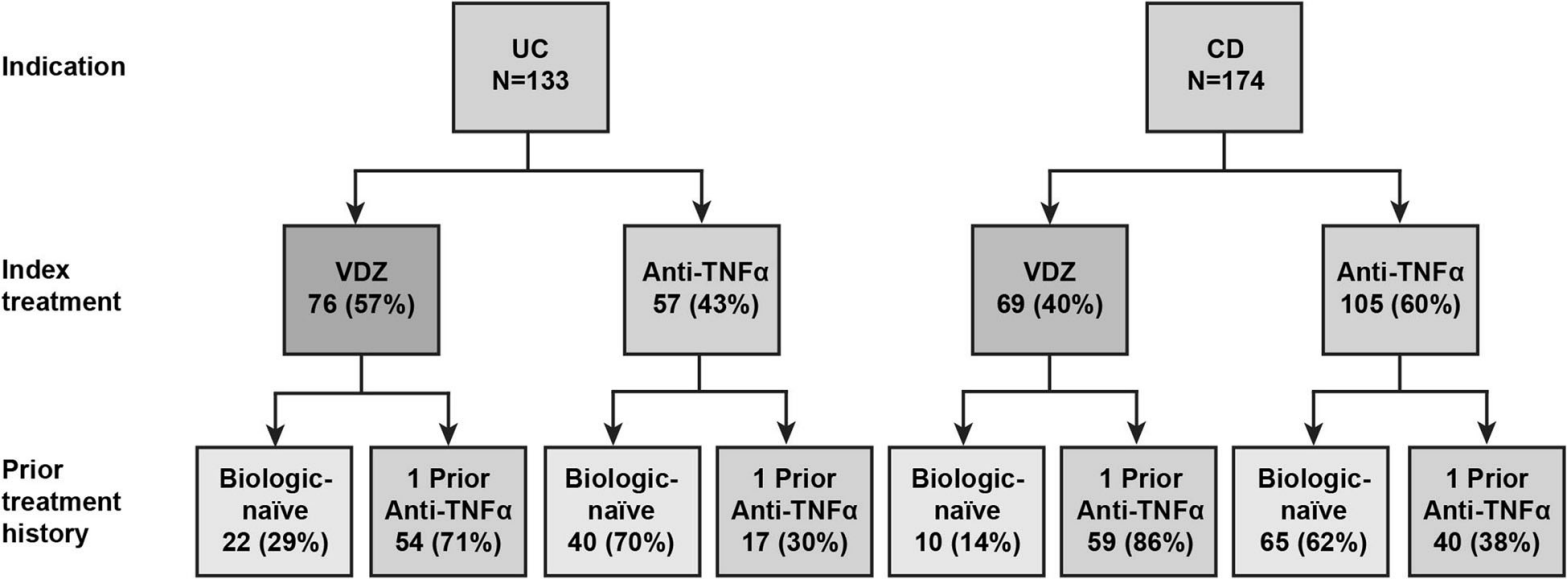
Study population and treatment cohorts. Anti-TNFα: anti-tumor necrosis factor alpha; CD: Crohn’s disease; UC: ulcerative colitis; VDZ: vedolizumab

A total of 145 patients with UC met the study inclusion criteria, of whom 133 were included in the full effectiveness analysis set. Of these, 76 patients (22 [29%] were biologic-naïve) initiated treatment with vedolizumab and 57 patients (40 [70%] were biologic-naïve) initiated treatment with an anti-TNFα (12 adalimumab, 14 golimumab, 31 infliximab [6 of these patients received an infliximab biosimilar]) (Fig. 1).

At baseline, UC patients treated with vedolizumab or anti-TNFα were 47% vs 58% female, median patient age was 39.5 vs 34.0 years, and median disease duration was 6.1 vs 4.7 years. In both treatment cohorts, most patients with known disease location had extensive colitis or pancolitis (65% on vedolizumab vs 51% on anti-TNFα). The mean (±SD) baseline total Mayo scores (6.0 [2.9] and 6.2 [2.9]) and the mean (±SD) partial Mayo scores (4.9 [2.6] and 5.4 [2.5]) were similar in the vedolizumab and anti-TNFα patient cohorts; however, a substantially larger proportion of vedolizumab compared with anti-TNFα patients had moderate to severe endoscopic disease (62% vs 39%; Table 1). Rectal bleeding (RB score ≥ 1) was present at baseline in 60% of vedolizumab and 66% of anti-TNFα patients, and ≥ 3 stools per day more than normal were experienced at baseline by 71% of vedolizumab and 82% of anti-TNFα patients (Table 1).

**Table 1:** Baseline demographics and clinical characteristics of patients with ulcerative colitis

Index Treatment group	VDZ	Anti-TNFα	VDZ	Anti-TNFα	VDZ	Anti-TNFα
Treatment history [Total N]	Biologic-naïve [*N* = 22]	Biologic-naïve [*N* = 40]	Prior anti-TNFα [*N* = 54]	Prior anti-TNFα [*N* = 17]	Total [*N* = 76]	Total [*N* = 57]
Female, %	36	60	52	53	47	58
Age at index, years, median (range)	46.5 (18–75)	33.0 (19–76)	35.0 (20–66)	41.0 (22–60)	39.5 (18–75)	34.0 (19–76)
UC duration, years, median (range) [n with available data]^a^	5.8 (0–29) [21]	4.2 (0–33) [40]	6.7 (1–47) [54]	6.7 (2–41) [17]	6.1 (0–47) [75]	4.7 (0–41) [57]
UC location, [n]	[21]	[35]	[45]	[12]	[66]	[47]
Ulcerative proctitis, %	0	9	9	8	6	9
Left-sided, %	29	40	29	42	29	40
Extensive colitis, %	10	11	9	17	9	13
Pancolitis, %	62	40	53	33	56	38
Prior IBD-related surgery, %	5	5	2	0	3	4
Total Mayo score, mean (SD) [n]	5.9 (3.2) [10]	5.7 (2.7) [16]	6.1 (2.8) [27]	7.7 (3.0) [6]	6.0 (2.9) [37]	6.2 (2.9) [22]
Partial Mayo score, mean (SD) [n]	4.8 (2.7) [12]	4.8 (2.5) [18]	4.9 (2.5) [35]	6.9 (2.1) [7]	4.9 (2.6) [47]	5.4 (2.5) [25]
Rectal bleeding score, [n]	[14]	[26]	[44]	[12]	[58]	[38]
0 (no blood seen), %	36	38	41	25	40	34
1 (streaks of blood with stool < half of time), %	36	23	30	17	31	21
2 (obvious blood with stool most of the time), %	14	27	23	25	21	26
3 (blood alone passes), %	14	12	7	33	9	18
Stool frequency score, [n]	[15]	[23]	[48]	[11]	[63]	[34]
0 (normal number of stools), %	7	4	17	0	14	3
1 (1–2 stools/day more than normal), %	33	22	8	0	14	15
2 (3–4 stools/day more than normal), %	13	22	19	9	17	18
3 (≥5 stools/day more than normal), %	47	52	56	91	54	65
Endoscopic score, [n]	[12]	[20]	[27]	[8]	[39]	[28]
0 (normal or inactive disease), %	25	30	11	38	15	32
1 (mild disease), %	33	35	19	13	23	29
2 (moderate disease), %	25	25	48	25	41	25
3 (severe disease), %	17	10	22	25	21	14
Corticosteroids in past 2 years, %	73	68	78	76	76	70
Aminosalicylates in past 2 years, %	86	70	78	71	80	70
Immunomodulators in past 2 years, %	55	58	43	59	46	58
Duration of previous anti-TNFα treatment, months, mean (SD), [n]	–	–	16.8 (16.9) [47]	19.5 (17.0) [16]	16.8 (16.9) [47]	19.5 (17.0) [16]
Duration from prior anti-TNFα discontinuation to index date, months, median (range), [n]	–	–	1.4 (0–32) [50]	1.2 (0–44) [17]	1.4 (0–32) [50]	1.2 (0–44) [17]
Index treatment, anti-TNFα type						
Infliximab originator, %	–	55	–	18	–	44
Infliximab biosimilar, %	–	15	–	0	–	11
Adalimumab, %	–	15	–	35	–	21
Golimumab, %	–	15	–	47	–	25
Concomitant therapy at index						
Corticosteroids, %	41	50	44	41	43	47
Immunomodulators, %	23	28	20	35	21	30
Aminosalicylates, %	59	50	56	53	57	51

*Anti-TNFα* Anti-tumor necrosis factor alpha, *IBD* Inflammatory bowel disease, *SD* Standard deviation, *UC* Ulcerative colitis, *VDZ* Vedolizumab

^a^Unless otherwise indicated, data from the full population were available for analysis

In the 2 years before index vedolizumab or anti-TNFα index treatment initiation, respectively, patients received corticosteroids (76% vs 70%), aminosalicylates (80% vs 70%), and immunomodulatory agents (46% vs 58%) (Table 1). Patients on vedolizumab or anti-TNFα with prior anti-TNFα treatment history had received their previous anti-TNFα for a mean (±SD) of 16.8 (16.9) and 19.5 (17.0) months, respectively, and had discontinued it for a median duration of 1.4 and 1.2 months prior to index, respectively (Table 1). Concomitant baseline medication use at index for patients receiving vedolizumab or anti-TNFα index treatment included corticosteroids (43% vs 47%), immunomodulators (21% vs 30%), and aminosalicylates (57% vs 51%), respectively.

#### CD patients

A total of 188 patients with CD met the inclusion criteria, of whom 174 were included in the full effectiveness analysis set. Overall, 69 patients (10 [14%] were biologic-naïve) initiated treatment with vedolizumab and 105 patients (65 [62%] were biologic-naïve) initiated treatment with an anti-TNFα (40 received adalimumab, 65 received infliximab [20 of these patients received an infliximab biosimilar]) (Fig. 1, Table 2).

**Table 2:** Baseline demographics and clinical characteristics of patients with Crohn’s disease

Index Treatment group	VDZ	Anti-TNFα	VDZ	Anti-TNFα	VDZ	Anti-TNFα
Treatment history [Total N]	Biologic-naïve [*N* = 10]	Biologic-naïve [*N* = 65]	Prior anti-TNFα [*N* = 59]	Prior anti-TNFα [*N*= 40]	Total [*N* = 69]	Total [*N* = 105]
Female, %	60	52	68	60	67	55
Age at index, years, median (range)	39.0 (21–70)	38.0 (18–72)	42.0 (20–73)	40.5 (21–58)	41.0 (20–73)	39.0 (18–72)
CD duration, years, median (range) [n with available data]^a^	8.2 (0–30) [10]	3.7 (0–50) [65]	10.1 (1–35) [58]	7.2 (0–33) [40]	9.8 (0–35) [68]	5.6 (0–50) [105]
CD location, [n]	[9]	[57]	[57]	[34]	[66]	[91]
Ileal, %	22	18	26	24	26	20
Colonic, %	33	28	23	15	24	23
Ileocolonic, %	44	54	51	62	50	57
Disease behavior, [n]	[9]	[60]	[52]	[34]	[61]	[94]
Non-stricturing, non-penetrating, %	44	65	67	38	64	55
Stricturing, %	44	22	21	41	25	29
Penetrating, %	11	13	12	21	11	16
Prior IBD-related surgery, %	20	35	42	45	39	39
HBI score, [n]	[3]	[10]	[10]	[13]	[13]	[23]
0–4 (remission), %	33	40	30	46	31	43
5–7 (mild activity), %	33	60	30	15	31	35
8–16 (moderate activity), %	33	0	40	38	38	22
≥ 17 (severe activity), %	0	0	0	0	0	0
Abdominal pain score, [n]	[5]	[46]	[36]	[22]	[41]	[68]
0 (none), %	20	39	25	41	24	40
1 (mild), %	0	20	19	18	17	19
2 (moderate), %	80	37	47	27	51	34
3 (severe), %	0	4	8	14	7	7
Liquid/soft stools per day, mean (SD) [n]	3.7 (2.5) [3]	3.6 (3.8) [35]	5.2 (4.9) [26]	3.8 (4.7) [21]	5.0 (4.7) [29]	3.7 (4.1) [56]
Endoscopic score, [n]	[6]	[39]	[30]	[26]	[36]	[65]
0 (normal or inactive disease), %	17	10	27	27	25	17
1 (mild disease), %	17	21	20	19	19	20
2 (moderate disease), %	33	46	20	23	22	37
3 (severe disease), %	33	23	33	31	33	26
Corticosteroids in past 2 years, %	60	69	58	53	58	63
Aminosalicylates in past 2 years, %	20	37	31	45	29	40
Immunomodulators in past 2 years, %	20	57	29	48	28	53
Duration of previous anti-TNFα treatment, months, mean (SD), [n]	–	–	31.6 (24.3) [52]	22.3 (20.7) [39]	31.6 (24.3) [52]	22.3 (20.7) [39]
Duration from prior anti-TNFα discontinuation to index date, months, median (range), [n]	–	–	1.1 (0–103) [57]	2.1 (0–57) [39]	1.1 (0–103) [57]	2.1 (0–57) [39]
Index treatment, anti-TNFα type						
Infliximab originator, %	–	42	–	45	–	43
Infliximab biosimilar, %	–	18	–	20	–	19
Adalimumab, %	–	40	–	35	–	38
Concomitant therapy at index						
Corticosteroids, %	20	25	29	28	28	26
Immunomodulators, %	10	19	8	18	9	18
Aminosalicylates, %	0	14	15	20	13	16

*Anti-TNFα* Anti-tumor necrosis factor alpha, *CD* Crohn’s disease, *HBI* Harvey Bradshaw Index, *IBD* Inflammatory bowel disease, *SD* Standard deviation, *VDZ* Vedolizumab

^a^Unless otherwise indicated, data from the full population were available for analysis

At baseline, CD patients treated with vedolizumab or anti-TNFα were 67% vs 55% female, median patient age was 41.0 vs 39.0 years, and median disease duration was 9.8 vs 5.6 years. Vedolizumab and anti-TNFα patients, respectively, had ileocolonic CD (50% vs 57%), stricturing (25% vs 29%), or penetrating disease (11% vs 16%) (Table 2). Moderate or severe disease activity (HBI ≥8) was recorded in 38% of vedolizumab and 22% of anti-TNFα patients, while moderate to severe endoscopic disease was present in 56 and 63% of patients, respectively (Table 2). Moderate to severe abdominal pain (AP score ≥ 2) was experienced at baseline by 59% of vedolizumab and 41% of anti-TNFα patients, while mean (±SD) number of liquid or very soft stools per day at baseline was 5.0 (4.7) for vedolizumab patients and 3.7 (4.1) for anti-TNFα patients (Table 2).

Crohn’s disease medications used within the 2 years before vedolizumab or anti-TNFα index treatment initiation, respectively, included corticosteroids (58% vs 63%), aminosalicylates (29% vs 40%), and immunomodulatory agents (28% vs 53%) (Table 2). Vedolizumab patients with prior anti-TNFα treatment experience had received their previous anti-TNFα for a mean (±SD) of 31.6 (24.3) months and had discontinued it a median of 1.1 months before index; anti-TNFα patients with prior anti-TNFα treatment history had received their previous anti-TNFα for a mean (±SD) of 22.3 (20.7) months and had discontinued it a median of 2.1 months before index (Table 2). Concomitant baseline medication use for patients receiving vedolizumab or anti-TNFα index treatment included corticosteroids (28% vs 26%), immunomodulators (9% vs 18%), and aminosalicylates (13% vs 16%), respectively.

### Clinical effectiveness in UC

#### Clinical remission

**Fig. 2:**
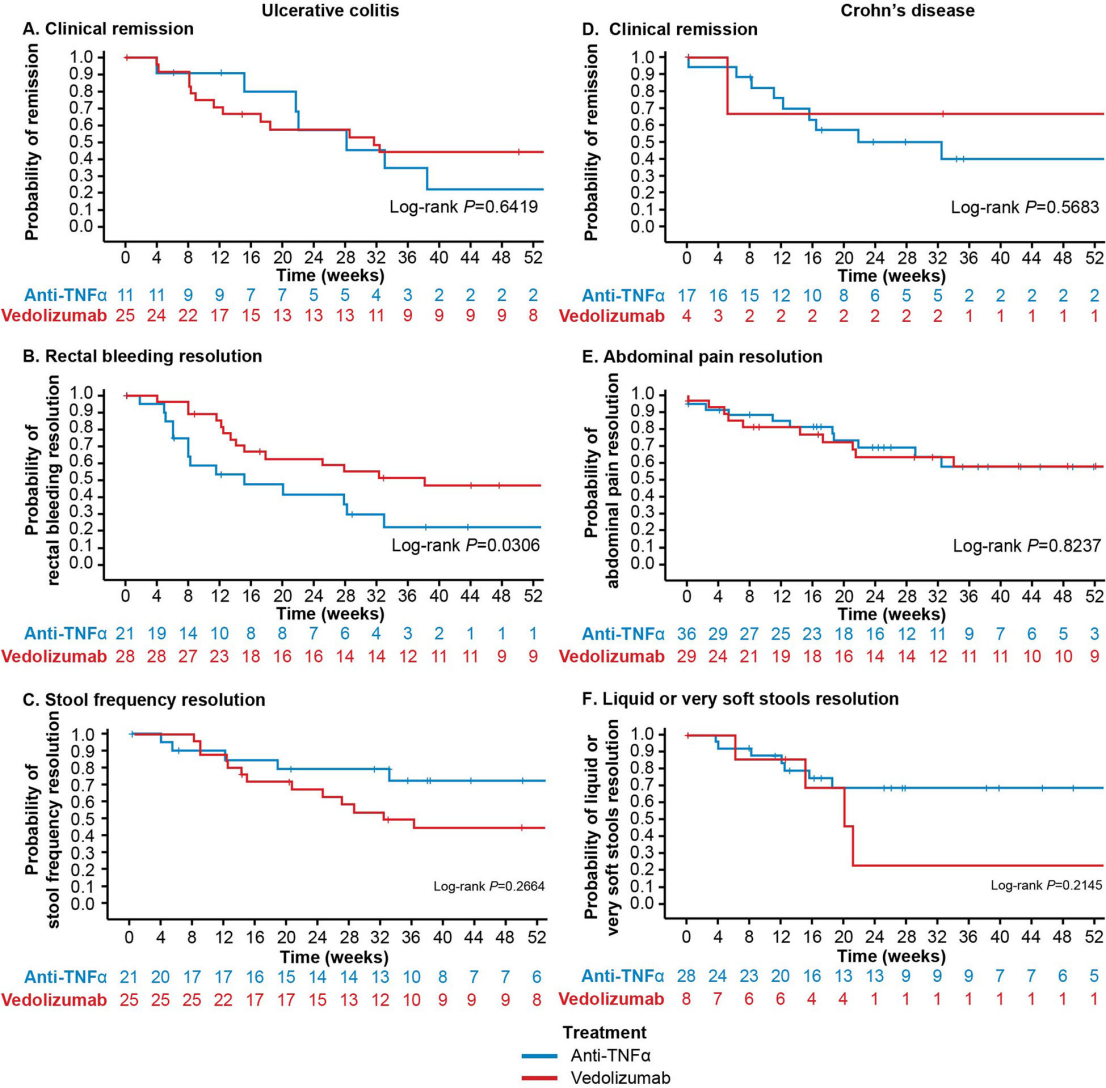
Time to first outcome by index treatment. Kaplan-Meier time to first outcome in patients with ulcerative colitis (panels **a-c**) or Crohn’s disease (panels **d-f**) by index treatment (vedolizumab vs anti-tumor necrosis factor alpha [anti-TNFα])

**Fig. 3:**
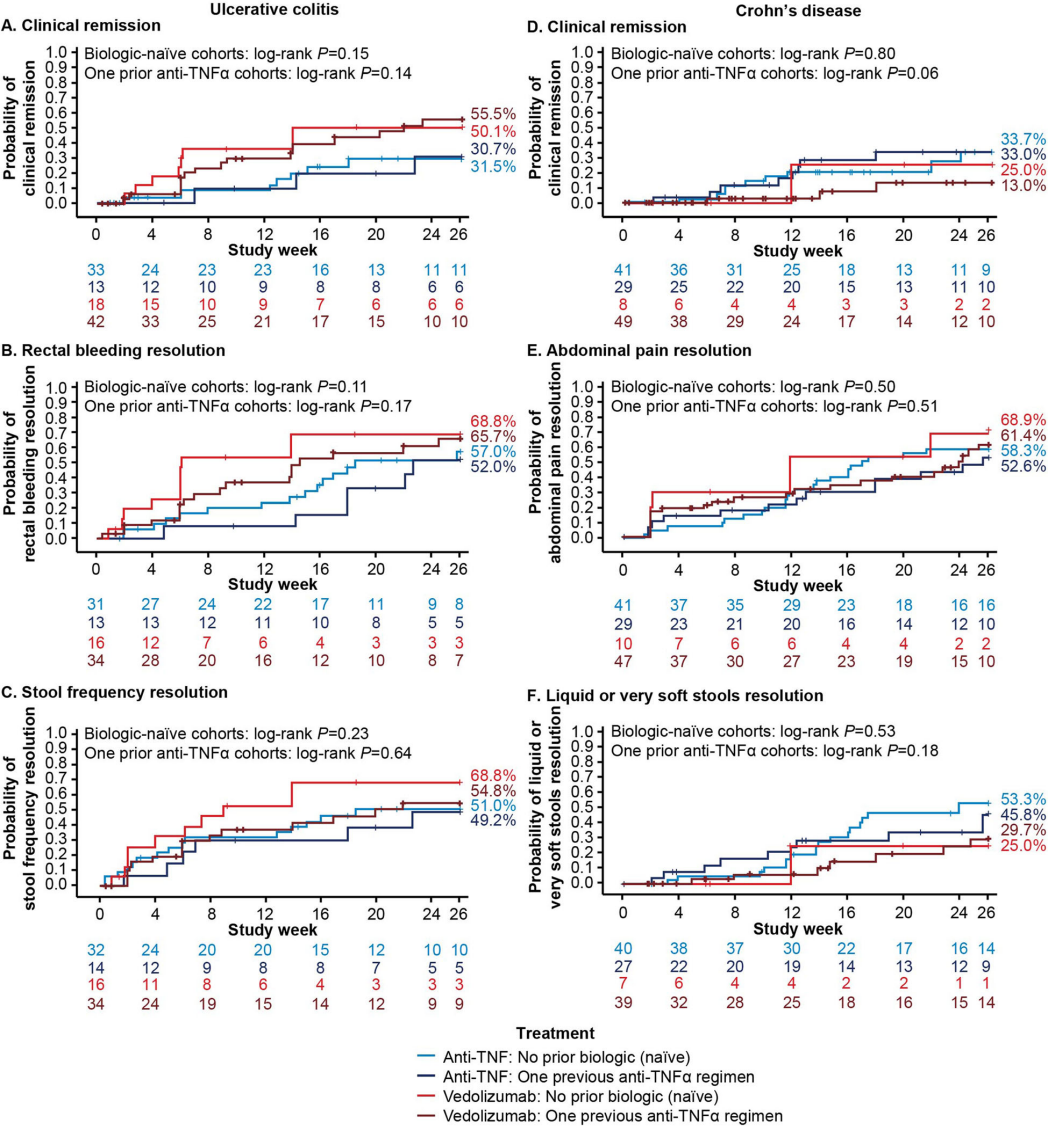
Time to first outcome by index treatment and prior biologic exposure. Kaplan-Meier time to first outcome in patients with ulcerative colitis (panels **a-c**) or Crohn’s disease (panels **d-f**) by index treatment (vedolizumab vs anti-tumor necrosis factor alpha [anti-TNFα]) and prior biologic exposure (biologic-naïve vs one previous anti-TNFα agent)

**Fig. 4:**
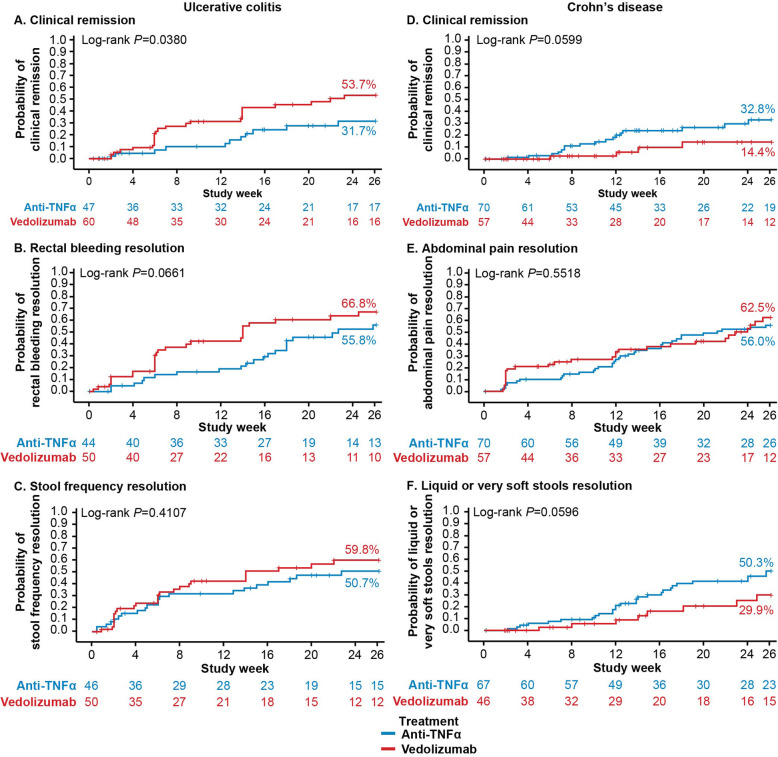
Duration of treatment outcomes by index treatment. Kaplan-Meier analysis of the duration of treatment outcomes in patients with ulcerative colitis (panels **a-c**) or Crohn’s disease (panels **d-f**) by index treatment (vedolizumab vs anti-tumor necrosis factor alpha [anti-TNFα])

The estimated cumulative rates of patients achieving CR by Week 26 were higher with vedolizumab treatment (53.7%) than with anti-TNFα treatment (31.7%) (Fig. 2). Clinical remission trends in the prior anti-TNFα exposure status sub-cohorts were consistent with the overall finding. In the biologic-naïve sub-cohort, estimated rates of patients achieving CR by Week 26 were 50.1% for vedolizumab and 31.5% for anti-TNFα. In the one prior anti-TNFα cohort, CR was estimated to have been achieved by 55.5% vs 30.7% of vedolizumab and anti-TNFα patients, respectively (Fig. 3). The median duration that CR was estimated to be sustained for was 31.7 weeks for vedolizumab vs 28.3 weeks for anti-TNFα (log-rank *P* = 0.64) (Fig. 4).

#### Rectal bleeding resolution

The estimated cumulative rates of patients achieving RB resolution by Week 26 were 66.8% with vedolizumab treatment vs 55.8% with anti-TNFα treatment (Fig. 2). As with CR, RB resolution trends in the prior anti-TNFα exposure status sub-cohorts were consistent with the overall finding. Among biologic-naïve patients, 66.8% of vedolizumab vs 57.0% of anti-TNFα patients were estimated to have achieved RB resolution by Week 26 (Fig. 3). Similarly, in the one prior anti-TNFα cohort, 65.7% of vedolizumab vs 52.0% of anti-TNFα patients achieved RB resolution by Week 26 (Fig. 3). The median duration of RB resolution was estimated to be significantly longer for vedolizumab vs anti-TNFα patients (38.1 vs 15.1 weeks, log-rank *P* = 0.03) (Fig. 4).

#### Stool frequency resolution

The estimated cumulative rates of patients achieving SF resolution by Week 26 were 59.8% with vedolizumab treatment vs 50.7% with anti-TNFα treatment (Fig. 2). Estimated rates of SF resolution were higher in biologic-naïve vedolizumab patients vs vedolizumab patients with prior anti-TNFα experience but were fairly consistent across sub-cohorts of anti-TNFα patients: among biologic-naïve patients, 66.8% of vedolizumab vs 51.0% of anti-TNFα patients were estimated to have achieved SF resolution by Week 26, whereas in the one prior anti-TNFα cohort, 54.8% of vedolizumab vs 49.2% of anti-TNFα patients achieved SF resolution by Week 26 (Fig. 3). The median duration of SF resolution was estimated to be 32.4 vs 86.3 weeks for vedolizumab and anti-TNFα patients, respectively (log-rank *P* = 0.27) (Fig. 4).

### Clinical effectiveness in CD

#### Clinical remission

The estimated cumulative rates of patients achieving clinical remission by Week 26 were 14.4% with vedolizumab vs 32.8% with anti-TNFα treatment (Fig. 2). Estimated rates of CR were higher in biologic-naïve vedolizumab patients vs vedolizumab patients with prior anti-TNFα experience but were consistent across sub-cohorts of anti-TNFα patients: in the biologic-naïve cohort, 25.0% of vedolizumab vs 33.7% of anti-TNFα patients were estimated to have achieved CR by Week 26 and 13.0% vs 33.0% in the one prior anti-TNFα cohort (Fig. 3). The median duration CR was estimated to be sustained for by anti-TNFα patients was 21.9 weeks (Fig. 4). In the small sample of vedolizumab patients who achieved CR by Week 26, median duration of CR could not be calculated.

#### Abdominal pain resolution

The estimated cumulative rates of patients achieving AP resolution by Week 26 were 62.5% with vedolizumab treatment vs 56.0% with anti-TNFα treatment (Fig. 2). Among both vedolizumab and anti-TNFα patients, estimated rates of AP resolution were slightly higher in biologic-naïve vs one prior anti-TNFα patients. Among biologic-naïve patients, 68.9% of vedolizumab vs 58.3% of anti-TNFα patients were estimated to have achieved AP resolution by Week 26 (Fig. 3). In the one prior anti-TNFα cohort, 61.4% of vedolizumab vs 52.6% of anti-TNFα patients were estimated to have achieved AP resolution by Week 26 (Fig. 3). The median duration of AP resolution was estimated to be > 52 weeks in all patients who achieved AP resolution by Week 26 (Fig. 4).

#### Liquid stools resolution

The estimated cumulative rates of patients achieving LS resolution by Week 26 were 29.9% with vedolizumab vs 50.3% with anti-TNFα treatment (Fig. 2). Among biologic-naïve patients, 25.0% of vedolizumab vs 53.3% of anti-TNFα patients were estimated to have achieved LS resolution by Week 26 (Fig. 3). Among patients with prior anti-TNFα exposure, 29.7% of vedolizumab vs 45.8% of anti-TNFα patients were estimated to have achieved LS resolution by Week 26 (Fig. 3). In patients who achieved LS resolution by Week 26, the median time to loss of LS resolution was estimated to be 20.1 weeks for vedolizumab patients and > 52 weeks for anti-TNFα patients (log-rank *P* = 0.21) (Fig. 4).

### Safety outcomes

#### UC patients

Treatment-related AEs occurred in 5.3% vs 7.0% of patients (4.0 vs 5.8 per 100 patient-years) and serious adverse events (SAEs) occurred in 13.2% vs 5.3% of patients (10.3 vs 4.3 per 100 patient-years) in the vedolizumab cohort vs anti-TNFα cohort, respectively. Among biologic-naïve patients, treatment-related AEs occurred in 4.5% vs 7.5% of patients and SAEs occurred in 4.5% vs 5.0% of patients in the vedolizumab cohort vs anti-TNFα cohort, respectively (Table 3). Infection was the only adverse event reported by > 5% of patients during vedolizumab treatment (6 patients; 7.9%). On anti-TNFα treatment > 5% of patients reported infection (8 patients; 14.0%), nausea (4 patients; 7.0%), anemia (4 patients; 7.0%), arthralgia (3 patients; 5.3%) and eczema (3 patients; 5.3%). There were no UC patient deaths during the study period.

**Table 3:** Summary of adverse events

**Patients with ulcerative colitis [*N*** **= 133]**						
**Index Treatment group**	**VDZ**	**Anti-TNFα**	**VDZ**	**Anti-TNFα**	**VDZ**	**Anti-TNFα**
**Treatment history**	**Biologic-naïve [N = 22]**	**Biologic-naïve [N = 40]**	**Prior anti-TNFα [N = 54]**	**Prior anti-TNFα [N = 17]**	**Total [N = 76]**	**Total [N = 57]**
Any AE						
Patients with event, n (%)	8 (36.4)	21 (52.5)	22 (40.7)	4 (23.5)	30 (39.5)	25 (43.9)
Patients with event per 100 patient-years, n (95% CI)	36.3 (15.7–71.6)	67.2 (41.6–102.7)	42.6 (26.7–64.5)	20.3 (5.5–52.1)	40.7 (27.5–58.1)	49.1 (31.8–72.4)
Treatment-related AE						
Patients with event, n (%)	1 (4.5)	3 (7.5)	3 (5.6)	1 (5.9)	4 (5.3)	4 (7.0)
Patients with event per 100 patient-years, n (95% CI)	3.4 (0.1–18.8)	6.4 (1.3–18.8)	4.3 (0.9–12.5)	4.4 (0.1–24.7)	4.0 (1.1–10.3)	5.8 (1.6–14.8)
Any SAE						
Patients with event, n (%)	1 (4.5)	2 (5.0)	9 (16.7)	1 (5.9)	10 (13.2)	3 (5.3)
Patients with event per 100 patient-years, n (95% CI)	3.5 (0.1–19.5)	4.2 (0.5–15.2)	13.2 (6.0–25.1)	4.4 (0.1–24.6)	10.3 (5.0–19.0)	4.3 (0.9–12.5)
Any AE resulting in hospitalization						
Patients with event, n (%)	1 (4.5)	1 (2.5)	8 (14.8)	1 (5.9)	9 (11.8)	2 (3.5)
Patients with event per 100 patient-years, n (95% CI)	3.5 (0.1–19.5)	2.1 (0.1–11.6)	11.6 (5.0–22.9)	4.4 (0.1–24.6)	9.3 (4.2–17.6)	2.8 (0.3–10.2)
**Patients with Crohn’s disease [*N*** **= 174]**						
**Index Treatment group**	**VDZ**	**Anti-TNFα**	**VDZ**	**Anti-TNFα**	**VDZ**	**Anti-TNFα**
**Treatment history**	**Biologic-naïve [N = 10]**	**Biologic-naïve [N = 65]**	**Prior anti-TNFα [N = 59]**	**Prior anti-TNFα [N = 40]**	**Total [N = 69]**	**Total [N = 105]**
Any AE						
Patients with event, n (%)	4 (40.0)	30 (46.2)	18 (30.5)	15 (37.5)	22 (31.9)	45 (42.9)
Patients with event per 100 patient-years, n (95% CI)	45.2 (12.3–115.8)	51.6 (34.8–73.6)	31.3 (18.5–49.4)	39.9 (22.3–65.8)	33.1 (20.8–50.1)	47.0 (34.3–62.9)
Treatment-related AE						
Patients with event, n (%)	1 (10.0)	16 (24.6)	5 (8.5)	4 (10.0)	6 (8.7)	20 (19.0)
Patients with event per 100 patient-years, n (95% CI)	7.7 (0.2–43.2)	21.7 (12.4–35.2)	7.2 (2.3–16.8)	8.0 (2.2–20.4)	7.3 (2.7–15.8)	16.1 (9.8–24.9)
Any SAE						
Patients with event, n (%)	1 (10.0)	9 (13.8)	6 (10.2)	3 (7.5)	7 (10.1)	12 (11.4)
Patients with event per 100 patient-years, n (95% CI)	8.3 (0.2–46.3)	11.3 (5.2–21.5)	8.5 (3.1–18.5)	5.9 (1.2–17.2)	8.5 (3.4–17.5)	9.2 (4.8–16.1)
Any AE resulting in hospitalization						
Patients with event, n (%)	1 (10)	5 (7.7)	3 (5.1)	3 (7.5)	4 (5.8)	8 (7.6)
Patients with event per 100 patient-years, n (95% CI)	8.3 (0.2–46.3)	6.1 (2.0–14.3)	4.1 (0.9–12.1)	5.9 (1.2–17.2)	4.7 (1.3–12.1)	6.0 (2.6–11.9)

*AE* Adverse event, *anti-TNFα* Anti-tumor necrosis factor alpha, *CI* Confidence interval, *SAE* Serious adverse event, *VDZ* Vedolizumab

#### CD patients

Treatment-related AEs occurred in 8.7% vs 19.0% of patients (7.3 vs 16.1 per 100 patient-years) and SAEs occurred in 10.1% vs 11.4% of patients (8.5 vs 9.2 per 100 patient-years) in the vedolizumab cohort vs anti-TNFα cohort, respectively. Among biologic-naïve patients, treatment-related AEs occurred in 10.0% vs 24.6% of patients and SAEs occurred in 10.0% vs 13.8% of patients in the vedolizumab cohort vs anti-TNFα cohort, respectively (Table 3). A hypersensitivity reaction was the only adverse event reported by > 5% of patients during vedolizumab treatment (4 patients; 5.8%) and a cutaneous reaction was the only adverse event reported by > 5% of patients during anti-TNFα treatment (6 patients; 5.7%). There were no CD patient deaths during the study period.

## Discussion

This study evaluated the real-world effectiveness and safety of vedolizumab and anti-TNFα treatments in patients with UC and CD in Germany during the time frame shortly after market authorization of vedolizumab. The proximity of the evaluation period of this study to the launch of vedolizumab in Germany may have imposed a selection bias in terms of physicians’ decision-making on whether to use vedolizumab as a first-line biologic and on whether to try a second anti-TNFα after an anti-TNFα failure. Vigilant AE reporting is also to be expected shortly after the approval of a new treatment. Within this context, our results provide a descriptive comparison of UC and CD real-world patient outcomes after vedolizumab and anti-TNFα treatment initiation and demonstrate the effectiveness and safety of vedolizumab and anti-TNFα in clinical practice.

At baseline, assessed descriptively, UC patients treated with vedolizumab and anti-TNFα in this study had a similar disease duration (median 6 vs 5 years). Disease activity assessed by mean total and partial Mayo scores was also similar between cohorts; however, a substantially larger proportion of vedolizumab compared with anti-TNFα patients had moderate to severe endoscopic disease (62% vs 39%). Also, a substantially lower proportion of vedolizumab patients were biologic-naïve (29% vs 70%). Within this population, our results indicate that by Week 26, estimated cumulative rates of CR were substantially higher for patients treated with vedolizumab compared with anti-TNFα. Similarly, symptom resolution rates were estimated to be slightly higher in vedolizumab compared with anti-TNFα patients. The median duration of RB resolution was significantly longer in patients on vedolizumab compared with anti-TNFα, whereas the duration of CR and SF was similar between the two treatment groups. Overall, similar or better outcomes were observed with vedolizumab than anti-TNFα despite a greater proportion of patients with moderate-to-severe disease in the vedolizumab cohorts.

In the CD cohorts, assessed descriptively, vedolizumab patients exhibited longer disease duration (median 10 vs 6 years), had more severe disease activity (38% vs 22% with HBI ≥8), and fewer were biologic-naïve (14% vs 62%) than anti-TNFα patients. Within this population, AP resolution was estimated to be achieved by more vedolizumab patients compared with anti-TNFα patients by Week 26 whereas CR and LS resolution outcomes were estimated to be achieved by more anti-TNFα compared with vedolizumab patients. Among CD patients, the median duration of outcomes assessed was similar among vedolizumab and anti-TNFα patients. Overall, similar outcomes were observed with vedolizumab and anti-TNFα cohorts despite a greater proportion of patients with moderate-to-severe disease in the vedolizumab cohorts.

A systematic review and meta-analysis of real-world IBD studies highlighted variable CR rates with vedolizumab treatment across geographic locations [19]. Pooled CR rates at 6 months were 39 and 26% in UC and CD patients, respectively (vs 54 and 14% at Week 26 in the current study). A recent post hoc analysis of data from phase 3 randomized controlled trials of vedolizumab vs placebo supports vedolizumab’s use as a first-line biologic in UC and CD patients [22]. The exploratory study demonstrated that substantial symptomatic improvement with vedolizumab treatment was achieved as early as Week 2, particularly in anti-TNFα–naïve patients compared with anti-TNFα–experienced patients [22].

Overall AEs in both treatment cohorts were as expected based on previously published studies [13, 14, 23]. Rates of treatment-related AEs in vedolizumab patients were comparable with anti-TNFα patients in UC (5.3% vs 7.0%); fewer treatment-related AEs were observed in vedolizumab patients versus anti-TNFα patients in CD (8.7% vs 19.0%). These results are consistent with the results of other studies comparing the real-world effectiveness and safety of vedolizumab and anti-TNFα for the treatment of IBD and may be related to the gut-selective mode of action of vedolizumab compared with the systemic immunosuppression of anti-TNFα [11–16, 24]. The SAE rates were notably lower (3 and 7% in UC and CD patients, respectively) in GEMINI 1 and 2 trials compared with our study (13.2 and 10.1% in UC and CD patients, respectively) [13, 14]. However, in a systematic review of real-world vedolizumab studies, SAE rates ranged from 0 to 13% [19], while a study with weighted pooled real-world SAE rates also showed a lower SAE rate of 8% [25]. It is likely that this variability is related to differences in the disease severity of patients at baseline.

The comparative effectiveness results of this study are also in line with published studies. A real-world comparison of the effectiveness of vedolizumab vs infliximab induction therapy among patients with moderately to severely active UC at a tertiary IBD center in the US revealed an overall numerically higher response rate with vedolizumab (78% vs 67%) [26]. Comparative effectiveness studies using propensity score-matched real-world data from the multicenter VICTORY Consortium found that, after accounting for measurable disease- and patient-specific characteristics that may affect biological effectiveness, UC patients treated with vedolizumab had significantly higher 12-month cumulative rates of CR compared with patients treated with an anti-TNFα [27]. The CD patients treated with vedolizumab had numerically (but not statistically significant) higher 12-month cumulative rates of CR compared with patients treated with an anti-TNFα [24]. In addition, for both UC and CD patients, safety profiles were improved with vedolizumab vs anti-TNFα (numerically lower rates of serious infections; significantly lower rates of SAEs) [28]. Our real-world study results were recently corroborated by results from VARSITY, a double-blind, head-to-head study of patients with UC treated with vedolizumab or adalimumab [29]. In VARSITY, vedolizumab was superior to adalimumab in achieving clinical remission (31.3% vs 22.5%) and endoscopic improvement (39.7% vs 27.7%) at Week 52; exposure-adjusted AE rates were also lower with vedolizumab versus adalimumab [29].

A growing body of clinical and real-world evidence suggests that biologic treatments may be more effective in UC and CD patients without prior biologic treatment history [13, 14, 16, 17, 22, 30]. In a prospective observational study evaluating the clinical benefit of vedolizumab over 1 year, CR rates were higher in biologic-naïve vs anti-TNFα–experienced patients (UC [55% vs 18%] and CD [33% vs 20%]) [17]. The results of the current study are aligned with these previous results, generally identifying better estimated outcomes in biologic-naïve vs anti-TNFα–experienced cohorts, independent of index treatment and indication.

This real-world chart review study had several limitations. As a retrospective study, data availability (to assess clinical effectiveness, safety and use of concomitant medications or treatments) was limited to what was recorded in the patient chart as part of routine clinical care. Missing data from patient records affected our ability to assess patients’ baseline disease activity, with some patients potentially in CR or having achieved symptom resolution at baseline but not left censored because their disease activity was unknown. Patients may also have achieved or lost CR or symptom resolution before the event being recorded in the chart, which could affect the KM estimates. Missing data may also mean that the rate of adverse events is underestimated. The distribution of missing data across treatment arms is unknown, and as such potential introduction of bias cannot be ruled out. Similarly, the introduction of bias based on treatment selection is possible as the analyses did not control for variability in patients’ baseline characteristics or baseline treatment dose schedules. Differences in baseline disease location and disease behavior may contribute to explaining variability in clinical effectiveness. Future studies evaluating comparative treatment outcomes should seek to control for disease severity and other patient characteristics at baseline; this was not feasible in this study due to small sample sizes, especially by sub-cohorts. We were also not able to assess an objective endpoint such as mucosal healing due to a lack of consistently recorded endoscopic data. Finally, small samples of patients in the sub-group analyses by prior anti-TNFα exposure limited our ability to interpret differences between groups.

Although recognizing the limitations of this study is important, this study also has a number of strengths, representing as it does the first retrospective, multicenter study in the German treatment context evaluating vedolizumab and anti-TNFα treatment outcomes in both biologic-naïve and anti-TNFα–refractory cohorts.

## Conclusions

The results of this study demonstrate that both vedolizumab and anti-TNFα agents are effective in achieving CR and symptom resolution in real-world patients with UC or CD.

## Supplementary information

**Additional file 1.** Study sites and affiliated ethics committee study approvals.

## Data Availability

The anonymized datasets used and/or analysed during the current study are available from the corresponding author on reasonable request.

## Abbreviations

AE: Adverse event
Anti-TNFα: Anti-tumor necrosis factor alpha
AP: Abdominal pain
CD: Crohn’s disease
CI: Confidence interval
CR: Clinical remission
EMA: European Medicines Agency
HBI: Harvey-Bradshaw Index
IBD: Inflammatory bowel disease
KM: Kaplan-Meier
LS: Loose stools
MAdCAM-1: Mucosal vascular addressin cell adhesion molecule 1
RB: Rectal bleeding
SAE: Serious adverse event
SD: Standard deviation
SF: Stool frequency
STRIDE: Selecting Therapeutic Targets in Inflammatory Bowel Disease
UC: Ulcerative colitis
VDZ: Vedolizumab
