# Supplementary material for: Real-world clinical effectiveness and safety of vedolizumab and anti-tumor necrosis factor alpha treatment in ulcerative colitis and Crohn’s disease patients: a German retrospective chart review
Source: BMC Gastroenterol. 2020 Jul 8;20:211. doi: 10.1186/s12876-020-01332-w (PMC7341567; doi:10.1186/s12876-020-01332-w)
Supplement: Supplementary file 1 — Additional file 1. Study sites and affiliated ethics committee study approvals. [file 12876_2020_1332_MOESM1_ESM.pdf]

## Additional File 1

## Study sites and affiliated ethics committee study approvals

| Site Number | Site Type | Site Name and Address | Ethics Committee Name and Address | Ethics Committee Reference Number |
|---|---|---|---|---|
| 1 | Office-Based Clinic (Private Practice) | Practice Dr. Mross<br><br>Ehrenfelsstrasse 47<br>10318 Berlin | LaGeSo<br>Office of Ethics Committee of state Berlin<br><br>Fehrbelliner Platz 1<br>10707 Berlin | No submission required (according to § 15 of professional code of conduct "Berufsordnung" of physician chamber Berlin, no submission of a non-interventional study to local EC is required, if a positive vote from another EC is available) |
| 2 | Office-Based Clinic (Private Practice) | Gastroenterology joint practice Herne<br><br>Wiescherstr. 20<br>44623 Herne | Ethics Committee of physician chamber Westfalen-Lippe and of Medical department of Westfaelische Wilhelms-University Muenster<br><br>Gartenstr. 210 - 214<br>48147 Muenster | 2016-124-f-S (approved on May 12, 2016) |
| 3 | Office-Based Clinic (Private Practice) | Practice for Gastroenterology<br><br>Burgstrasse 61<br>69121 Heidelberg | Ethics Committee at state physician chamber Baden-Wuerttemberg<br><br>Jahnstraße 40<br>70597 Stuttgart | F-2016-024 (approved on April 26, 2016) |
| 4 | Office-Based Clinic (Private Practice) | Gastroenterology at Bayerischer Platz<br><br>Innsbrucker Str. 58<br>10825 Berlin | LaGeSo<br>Office of Ethics Committee of State Berlin Berlin<br><br>Fehrbelliner Platz 1<br>10707 Berlin | No submission required (according to § 15 of professional code of conduct "Berufsordnung" of physician chamber Berlin, no submission of a non-interventional study to local EC is required, if a positive vote from another EC is available) |
| 5 | Office-Based Clinic (Private Practice) | Gastroenterology Opernstrasse<br><br>Opernstrasse 9<br>34117 Kassel | Ethics Committee of State Physician Chamber Hessen<br><br>Im Vogelsgesang 3<br>60488 Frankfurt | III/1/ewa MC 106/2016 (approved on June 09, 2016) |
| 6 | Office-Based Clinic (Private Practice) | Internistic joint practice Oldenburg<br><br>Neue Donnerschweer Str. 30<br>26123 Oldenburg | Ethics Committee of Physician Chamber Niedersachsen<br><br>Berliner Allee 20<br>30175 Hannover | Grae/097/2016 (approved on May 25, 2016) |

| | | | | |
|---|---|---|---|---|
| 7 | Office-Based Clinic (Private Practice) | Gastro-Practice Wiesbaden<br><br>Langenbeckplatz 2<br>65189 Wiesbaden | Ethics Committee of state physician chamber Hessen<br><br>Im Vogelsgesang 3<br>60488 Frankfurt | III/1/ewa MC 106/2016 (approved on June 09, 2016) |
| 8 | Office-Based Clinic (Private Practice) | Practice Dr. med. B. Buendgens<br><br>Marienstrasse 47-51<br>52249 Eschweiler | Ethics Committee of Physician Chamber Nordrhein<br><br>Tersteegenstrasse 9<br>40474 Duesseldorf | 2016098 (approved on May 23, 2016) |
| 9 | Office-Based Clinic (Private Practice) | MVZ Dachau<br>Center for Internal Medicine and for General Medicine<br><br>Muenchner Str. 64<br>85221 Dachau | Ethics Committee of Bavarian State Physician Chamber<br><br>Mühlbaurstrasse 16<br>81677 Munich | No submission required (according to § 15 of professional code of conduct "Berufsordnung" of state physician chamber Bavaria, no submission of a non-interventional study to local EC is required, if a positive vote from another EC is available) |
| 10 | University Hospital | University clinic Muenster<br>Medizinische Klinik B<br><br>Albert-Schweitzer-Campus 1<br>48149 Münster | Ethics Committee of physician chamber Westfalen-Lippe and of Medical department of Westfaelische Wilhelms-University Muenster<br><br>Gartenstr. 210 - 214<br>48147 Muenster | 2016-124-f-S (approved on May 24, 2016) |
| 11 | University Hospital | Clinic of University Munich<br>Medical Clinic II, CED-Ambulance<br><br>Marchioninistr. 15<br>81377 Munich | Ethics Committee of Medical Department of Ludwig-Maximilians University Munich<br><br>Pettenkoferstr. 8 IV, Zi. I 4.07 — I 4.10<br>80336 Munich | 333-16 (approved on June 06, 2016) |
| 12 | Office-Based Clinic (Private Practice) | Gastro Campus Research gBR An der Germania<br><br>Brauerei 6<br>48159 Muenster | Ethics Committee of physician chamber Westfalen-Lippe and of Medical department of Westfaelische Wilhelms-University Muenster<br><br>Gartenstr. 210 - 214<br>48147 Muenster | 2016-124-f-S (approved on June 06, 2016) |
| 13 | Research Unit | Hamburgisches Forschungsinstitut fuer CED HaFCED e.K. second floor<br><br>Christoph-Probst-Weg 4<br>20251 Hamburg | Ethics Committee of Physician Chamber Hamburg<br><br>Weidestraße 122 b<br>22083 Hamburg | MC-176/16 (approved on June 22, 2016) |

| | | | | |
|---|---|---|---|---|
| 14 | University Hospital | University Clinic Magdeburg A.oe.R.<br>Clinic for Gastroenterology, Hepatology and Infectology<br><br>Leipziger Str. 44<br>39120 Magdeburg | Ethics Committee of Otto-von-Guericke-University at Medical Department and at University Clinic Magdeburg A. oe. R.<br><br>Leipziger Strasse 44<br>39120 Magdeburg | 75/16 (approved on June 06, 2016) |
| 15 | University Hospital | Charite University Medicine Berlin<br>Medical Clinic with focus on Gastroenterology and Hepatology<br><br>Augustenburger Platz 1<br>13353 Berlin | Ethics Committee of Medical Department of Charité University Medicine Berlin<br><br>Charitéplatz 1<br>10117 Berlin | No submission required (according to § 15 of professional code of conduct "Berufsordnung"of physician chamber Berlin, no submission of a non-interventional study to local EC is required, if a positive vote from another EC is available) |
